# The role of mitochondrial function in the pathogenesis of diabetes

**DOI:** 10.3389/fendo.2025.1607641

**Published:** 2025-08-08

**Authors:** Bo Qin, Yiying Wang, Jinlong Ding

**Affiliations:** ^1^ Shaoxing Maternity and Child Health Care Hospital, Shaoxing, China; ^2^ Maternity and Child Health Care Affiliated Hospital, Shaoxing University, Shaoxing, China

**Keywords:** mitochondrial function (MF), diabetes mellitus (DM), energy metabolism (EM), oxidative stress (OS), insulin sensitivity (IS)

## Abstract

Diabetes mellitus is a chronic metabolic disease characterized by abnormally elevated blood sugar levels due to insulin deficiency or insulin resistance, ultimately leading to various serious complications. In this review, we highlighted the significance of mitochondrial functionality in diabetes, concentrating on elements such as mitochondrial energy metabolism, oxidative stress, and their interplay with insulin sensitivity. Mitochondria are essential organelles that are crucial for energy production and integral to cellular metabolic processes. Recent investigations have underscored the role of mitochondrial dysfunction in the advancement of diabetes, uncovering fundamental mechanisms that lead to insulin resistance and elevated blood glucose levels. Present study amalgamates insights from contemporary studies, emphasizing the criticality of mitochondrial integrity in the occurrence of diabetes and its promise as a target for therapeutic intervention. By clarifying these associations, we aspire to offer novel perspectives and pathways for the prevention and management of diabetes.

## 1 Introduction

Diabetes mellitus (DM) represents a multifaceted metabolic condition marked by persistent hyperglycemia, which arises from deficiencies in either insulin secretion, insulin action, or a combination of both. The global incidence of DM has escalated to epidemic levels, impacting millions of individuals and resulting in considerable morbidity and mortality due to its associated complications, including cardiovascular diseases, nephropathy, neuropathy, retinopathy, etc. The World Health Organization (WHO) estimates that the prevalence of DM has been steadily increasing, approximately 578 million people will be affected by 2030. DM has been classified into type 1 diabetes (T1DM), type 2 diabetes mellitus (T2DM), gestational diabetes mellitus (GDM), and other types. Adiposity, insulin resistance in skeletal muscle, and decreased insulin production by pancreatic β cells which are responsible for synthesizing and secreting insulin, were the most important principal diabetogenic factors (1). The etiology of DM is intricate, encompassing a range of genetic, environmental, and lifestyle influences. Many factors, such as genetic defects, oxidative stress, metabolic abnormalities, environmental toxins and aging, can damage mitochondrial function and affect insulin synthesis, secretion and release. Dysfunction of lysosomes which are effector organelles of autophagic degradation is also a cause or aggravating element in DM and its complications (2). In both T1DM and T2DM, pancreatic β-cell survival and function are impaired more or less. It also has a lot to do with dysfunction in insulin-sensing hepatic, muscle, and adipose tissues as well as immune cells. Furthermore, mitochondria function and structure was a vital determinant of metabolic health across various tissues mentioned above. Recent investigations have underscored the pivotal role of mitochondrial dysfunction in the onset and advancement of DM, indicating that compromised mitochondrial performance may serve as a fundamental mechanism connecting various risk factors to the disease.

Mitochondria, double-membrane organelles, commonly known as the cell’s energy factories, are crucial for cellular energy metabolism, as they synthesize adenosine triphosphate (ATP) via oxidative phosphorylation. They are derived from α−proteobacteria which were engulfed by the precursor of modern eukaryotic cells before evolving as endosymbionts in ancient times. In addition to primary role in energy production, mitochondria participate in numerous vital cellular functions, play a core role in cell fate decisions via cellular energy production, reactive oxygen species (ROS) production, calcium (Ca^2+^) regulation, metabolic synthesis, immune signaling and programmed cell death (3). In addition to its own genome and the encoded proteins, mitochondrial function is mainly regulated and controlled by the nuclear-encoded genes via anterograde signals, mitochondrial biogenesis and activity will be regulated subsequently to adapt to cellular needs. From this, it follows that, mitochondrial-to-nuclear talk by altered levels of mitochondrial metabolites or stress signals causes various epigenetic changes, facilitating efforts to maintain homeostasis and health (4). Mitochondria are integral to pancreatic β-cells which are relatively metabolically active. The multifaceted portrayal of mitochondria in β-cells encompasses mitochondrial bioenergetics and metabolism, proton leak, Ca^2+^, structural integrity, dynamics, mitochondrial autophagy (mitophagy), etc. In relation to diabetes, mitochondrial impairment can lead to heightened oxidative stress, which aggravates insulin resistance and drives the advancement of diabetic complications. For example, research has indicated that mitochondrial dysfunction within pancreatic β-cells hinders insulin secretion and intensifies β-cell apoptosis, thereby disrupting glucose homeostasis (5).

**Figure 1:**
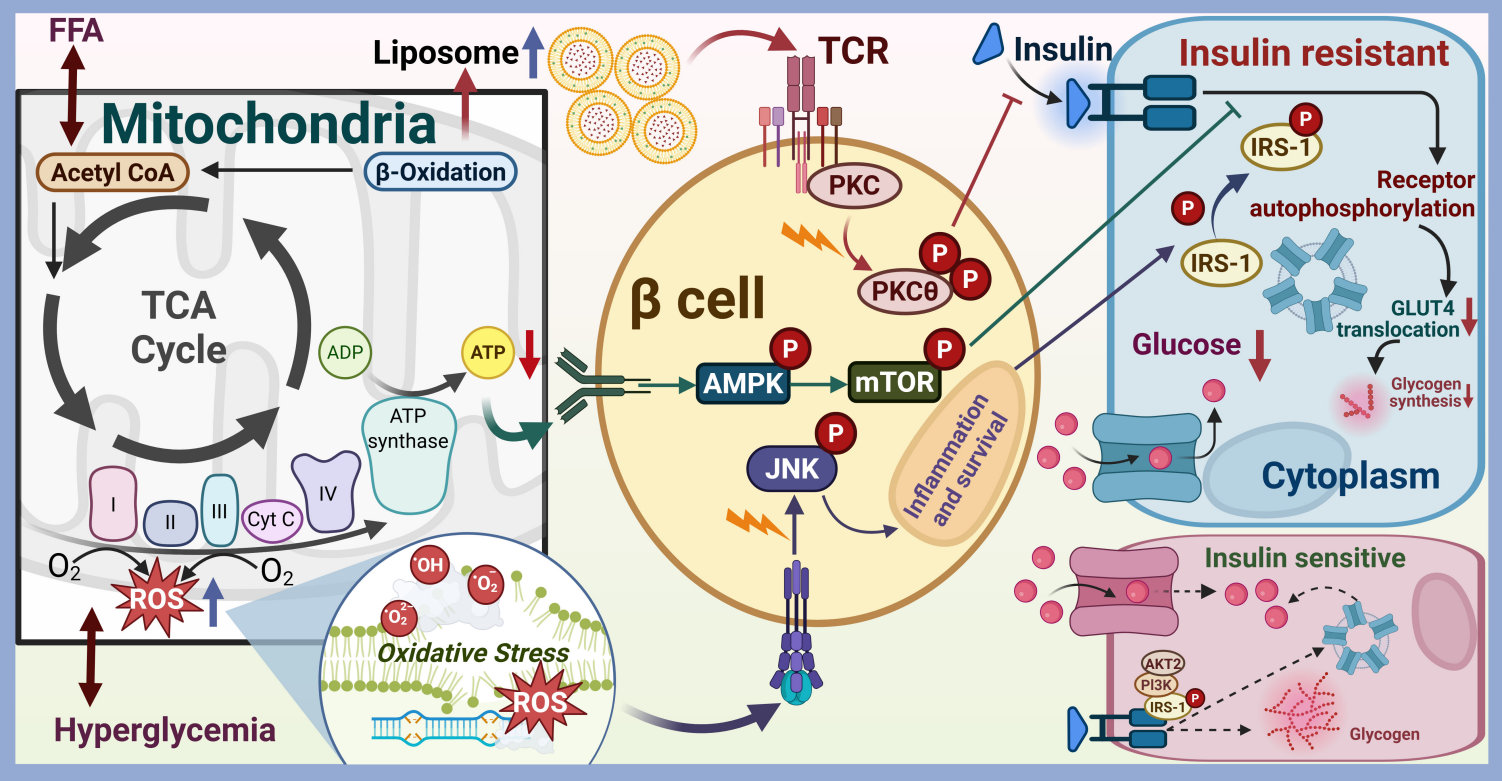
The pathway through which mitochondrial dysfunction leads to insulin resistance. Mitochondria are the primary organelles for cellular energy (ATP) production via oxidative phosphorylation (OXPHOS). Mitochondrial dysfunction (reduced biogenesis, impaired ETC function, decreased β-oxidation capacity, altered dynamics, increased ROS) in insulin-sensitive tissues (skeletal muscle, liver, adipose tissue) disrupts metabolic homeostasis, leading to insulin resistance.

Furthermore, the association between mitochondrial function and insulin resistance is especially significant in skeletal muscle and liver tissues, which play a vital role in sustaining whole-body glucose regulation. Thereat, mitochondrial dysfunction correlates with modifications in fatty acid and glucose metabolism, leading to insulin resistance (6, 7). As shown in **Figure 1**, the pathway through which mitochondrial dysfunction leads to insulin resistance was skeletonized. Lipid accumulation in cells can manifest in various forms, primarily characterized by the presence of distinct lipid metabolites such as fatty acids, diacylglycerol (DAG), ceramides, and lysophosphatidylcholine (lysoPC). Each of these metabolites plays a crucial role in cellular metabolism and signaling pathways. For instance, fatty acids, which are the primary components of triglycerides, can be stored in adipocytes or utilized for energy production through β-oxidation. However, excessive accumulation of fatty acids can lead to lipotoxicity, which is associated with insulin resistance and metabolic disorders. DAG, another significant lipid intermediate, is generated during the breakdown of triglycerides and phospholipids. Elevated levels of DAG have been implicated in the activation of protein kinase C (PKC), which can interfere with insulin signaling pathways, thereby contributing to insulin resistance and the development of T2DM (8–10). Ceramides, sphingolipid metabolites, are known to accumulate in various tissues, particularly in the liver and skeletal muscle, under conditions of metabolic stress. They have been shown to impair insulin signaling by promoting inflammation and oxidative stress, which further exacerbates insulin resistance (11, 12). Ceramides could directly inhibit Akt/PKB activation and translocation, activate protein phosphatases that dephosphorylate/inactivate Akt, and promote ER Stress and inflammation. Mitochondrial dysfunction can increase ROS production and oxidative stress, ETC impairment increases electron leak, generating excess superoxide (O_2_^-^) and other ROS. Antioxidant defenses, such as SOD and glutathione, may be overwhelmed, lipids, proteins and DNA will be damaged in the following. The accumulation of ceramides is particularly concerning as it correlates with the severity of metabolic diseases, including non-alcoholic fatty liver disease (NAFLD) and T2DM (13). ROS activate stress-sensitive serine/threonine kinases, such as c-Jun N-terminal Kinase (JNK), IκB Kinase β (IKKβ) and IκB Kinase β (IKKβ). JNK can phosphorylate IRS-1 on inhibitory serine residues, impairing insulin signaling. IKKβ Activates the transcription factor NF-κB, inducing expression of pro-inflammatory cytokines, such as TNFα, IL-1β and IL-6. p38 MAP Kinase could phosphorylate IRS proteins negatively. ROS can also directly oxidize and damage key insulin signaling molecules including insulin receptor, IRS and Akt (14). Mitochondrial damage which will release DAMPs such as mtDNA and cardiolipin, can activate innate immune sensors including TLRs and NLRP3 inflammasome. Chronic low-grade inflammation will result in production and secretion of pro-inflammatory cytokines including TNFα, IL-1β, IL-6 and MCP-1, which can activate JNK and IKKβ/NF-κB pathways within insulin-sensitive cells, leading to inhibitory serine phosphorylation of IRS proteins. They also can downregulate expression of insulin signaling components, promote adipose tissue lipolysis, increasing circulating FFAs and further exacerbating lipotoxicity (15). Mitochondrial dysfunction can lead to abnormal energy metabolism and insufficient ATP production. Increased AMP/ATP ratio activates AMP-activated Protein Kinase (AMPK). While acute AMPK activation promotes glucose uptake and FA oxidation, chronic activation in the context of mitochondrial dysfunction may contribute to insulin resistance in specific tissues by suppressing anabolic processes and potentially influencing gluconeogenesis regulation (16). Additionally, lysoPC, a product of phospholipid metabolism, has been associated with inflammation and cellular stress responses. Elevated levels of lysoPC can disrupt membrane integrity and contribute to the pathogenesis of metabolic disorders (12, 17). These pathways form a reinforcing network, where mitochondrial dysfunction initiates a cascade of lipotoxicity, oxidative stress, and inflammation that directly disrupts insulin signal transduction, primarily through inhibitory serine phosphorylation of IRS proteins, leading to systemic insulin resistance and the development of T2DM.

The dynamic nature of lipid accumulation and its metabolic intermediates is influenced by various factors, including dietary habits, physical activity, and genetic predispositions. High-fat diets have been shown to increase the levels of these lipid metabolites, leading to significant alterations in cellular metabolism and insulin sensitivity. Furthermore, the localization of these metabolites within different cellular compartments can dictate their functional roles. DAG is primarily found in the cytosolic compartment and can rapidly participate in signaling cascades, while ceramides can accumulate in lipid rafts, affecting membrane fluidity and protein interactions (18, 19). Numerous studies have shown that the capacity for mitochondrial respiration diminishes as diabetes advances, whereas interventions such as physical exercise and pharmacological therapies can enhance mitochondrial functionality and promote metabolic health (20). Insulin sensitivity, glucose tolerance and long-term glucose control were improved following exercise training interventions (21). Appreciating this significance, the potential for addressing mitochondrial dysfunction as a therapeutic approach in the management of diabetes was highlighted in this review. The involvement of mitochondria in the development of diabetes transcends mere energy metabolism. The dynamics of mitochondria, encompassing processes of fission and fusion, are essential for preserving mitochondrial integrity and functionality. Disruptions in these processes can result in the accumulation of impaired mitochondria, which can intensify cellular stress and inflammation. Mitophagy is an important mitochondrial quality control process in cells and a special autophagy phenomenon, which is involved in T2DM development by protecting pancreatic β-cells from insulin resistance (22). Damaged or redundant mitochondria can be selectively removed by autophagic lysosome, which is crucial to maintain cell stability and survival under stress (1). Changes in autophagy play a vital role in the development and control of DM and its complications. Metabolic overload at the mitochondrial level contributes to insulin resistance and pancreatic β-cell dysfunction primarily through incomplete β-oxidation of fatty acids. This inefficiency leads to the accumulation of toxic lipid intermediates, such as ceramides, which can further impair mitochondrial structure and function, exacerbating metabolic dysfunction (23). Mitophagy eliminates this vicious cycle of oxidative stress and mitochondrial damage, and thus counteracts pathogenic processes. Autophagy also mediates exercise-induced increases in muscle glucose uptake and protects β cells against ER stress in diabetogenic conditions. Dysfunctional mitophagy, responsible for the selective degradation of damaged mitochondria, has been linked to the onset of diabetic complications, such as diabetic cardiomyopathy and retinopathy (24). Transcription factor EB (TFEB), a major regulator of the autophagic lysosomal pathway which is associated with T2DM progression, can drive autophagy and lysosomal gene expression (25). Being drawn into many aspects mentioned above, mitophagy appears to be an attractive target for therapeutic interventions against not only diabetes but also obesity. Therefore, enhancing mitophagy may serve as a promising therapeutic method to alleviate the impact of DM. In addition to its direct metabolic effects, mitochondrial dysfunction is also associated with the inflammatory processes linked to diabetes. Mitochondria, regarded as a key regulator of inflammation, ROS formation causes opening of the mitochondrial permeability transition pore (mPTP), which chronically leads to the disruption of mitochondria with subsequent unspecific release of oxidized mtDNA, a damage-associated molecular pattern (DAMP) that activate inflammatory pathways, thereby contributing to the chronic low-grade inflammation characteristic of diabetes (26). This inflammatory environment further exacerbates insulin resistance and the progression of diabetic complications, underscoring the interrelatedness of mitochondrial function, inflammation, and metabolic dysregulation. Redox activation of immune cells and the subsequent excitation of the phagocytic NOX2 are efficiently striked by mitochondrial O_2_^˙-^/H_2_O_2_ formation via redox cross talk, which is key to the activation, recruitment, and infiltration of myelomonocytic and T cells (27).

Mitochondria-associated membranes (MAMs), ER-mitochondria contact sites between these two organelles, serve as a platform for autophagosomes and mitophagosomes formation, and different cellular processes regulation, many proteins that regulate the autophagy process are found to be localized there. The inositol 1, 4, 5-triphosphate receptors (IP3R) channels mediate the release of Ca^2+^ from ER to cytoplasm. Mitofusin, existing in two different isoforms-Mfn1 and Mfn2, are GTPases and located at the outer mitochondrial membrane (OMM) and mediate the mitochondrial fusion process. Mitochondrial fission 1 protein, a Drp1 adapter protein, presented on OMM and participated in the mitochondrial fission process (28). ORP5 and ORP8, oxysterol-binding protein (OSBP)-related proteins family (ORP) proteins, mediate the transport of PI(4)P and phosphatidyl serine (PS) from plasma membrane to ER, and involved in sterol homeostasis and vesicular trafficking (29). Being exposed to high levels of circulating nutrients, cellular stress stimulates autophagy in β-cells. Increase in autophagic flux can resolve the cellular stress and allowing maintenance of β-cell function and cellular survival. However, if the autophagy capacity is compromised triggered by some kind of pathological factor (genetic polymorphisms/alterations, advanced aging, etc.), cellular stress pathways will be enhanced via ER stress, oxidative stress, islet amyloid polypeptide (IAPP) deposition, inflammation, etc. Eventually it leads to β-cell dysfunction, dedifferentiation, cellular demise, peripheral insulin resistance, T2D will ensue if the dysfunctional/depleted β-cells fail to compensate. Autophagic insufficiency may occur secondary to the cellular stress, which drives lysosomal dysfunction, leading to deregulation of autophagic flux and failure of the pathway.

Given the pivotal role of mitochondria in the pathogenesis of diabetes, current research endeavors are aimed at clarifying the specific molecular mechanisms that underlie mitochondrial dysfunction and its role in insulin resistance and β-cell impairment. Moreover, the creation of innovative therapeutic strategies designed to restore mitochondrial function, such as mitochondria-targeted antioxidants, agents that stimulate mitochondrial biogenesis, and compounds that modulate mitochondrial dynamics, holds potential for enhancing outcomes in diabetic patients (30). In summary, understanding the multifaceted roles of mitochondria in the pathogenesis of diabetes provides valuable insights into prospective therapeutic strategies for this increasingly prevalent condition.

## 2 Mechanisms of mitochondrial energy metabolism

### 2.1 Function of the mitochondrial respiratory chain

Mitochondria are commonly designated as the cell’s powerhouses due to their essential involvement in energy metabolism, which encompasses a range of biochemical processes. The processes that govern mitochondrial energy metabolism are intricate and encompass several pathways that unify cellular respiration, ATP production, and the interactions with various metabolic substrates. This section will explore the sophisticated mechanisms of mitochondrial energy metabolism, with particular emphasis on the mitochondrial respiratory chain, the correlation between ATP synthesis and energy metabolism, as well as the interactions between mitochondria and carbohydrate metabolism.

The mitochondrial respiratory chain, frequently referred to as the electron transport chain (ETC), comprises a series of protein complexes embedded in the inner mitochondrial membrane. This chain plays a pivotal role in the oxidative phosphorylation process, a mechanism that facilitates ATP production through the transfer of electrons from electron donors to electron acceptors via redox reactions. The ETC is organized into four principal complexes (I-IV) and includes two mobile electron carriers, namely ubiquinone (coenzyme Q) and cytochrome c. As electrons traverse these complexes, protons (H^+^) are translocated from the mitochondrial matrix into the intermembrane space, thereby establishing a proton gradient across the inner mitochondrial membrane. This electrochemical gradient is subsequently harnessed by ATP synthase (complex V) to convert adenosine diphosphate (ADP) and inorganic phosphate (Pi) into ATP (23, 31) Mitochondrial dysfunction is implicated in a spectrum of metabolic disorders, such as diabetes and cardiovascular diseases. Compromised mitochondrial function heightens the vulnerability of myocardial tissue to ischemia-reperfusion injury, underscoring the significance of mitochondrial integrity in sustaining cardiac health. Furthermore, the equilibrium between mitochondrial fission and fusion is essential for preserving mitochondrial functionality and cellular homeostasis. An imbalance in these dynamic processes can lead to mitochondrial fragmentation, a phenomenon frequently observed in diabetic states, which correlates with heightened oxidative stress and increased rates of apoptosis (32).

### 2.2 Relationship between ATP synthesis and energy metabolism

ATP synthesis is fundamental to the metabolism of cellular energy, serving as the key energy currency that powers a multitude of cellular activities. The intricate relationship between ATP synthesis and energy metabolism is subject to precise regulation and is influenced by various factors, such as the availability of substrates, the functionality of mitochondria, and the energy demands of the cell. Mitochondria are crucial in this context, as they link the oxidation of nutrients to ATP generation through the process of oxidative phosphorylation. Mitochondrial dysfunction emerges as a critical factor contributing to the attenuation of ATP synthesis. Research has indicated that pancreatic β-cells demonstrate diminished ATP production under diabetic conditions, which adversely affects insulin secretion and the regulation of glucose levels (5). Additionally, the interaction between mitochondrial bioenergetics and cellular signaling pathways is vital for sustaining metabolic health. Mitochondria not only synthesize ATP but also generate reactive oxygen species (ROS) as byproducts during oxidative phosphorylation. While modest amounts of ROS can act as signaling molecules that facilitate adaptive responses, excessive ROS generation can induce oxidative stress and cellular injury, thereby worsening metabolic diseases (32). Furthermore, the regulation of ATP synthesis is contingent upon the availability of substrates like glucose and fatty acids. In conditions characterized by insulin resistance, the capacity of cells to utilize glucose for ATP generation is compromised, prompting a shift towards alternative energy substrates, such as fatty acid oxidation. This metabolic transition is frequently linked to augmented mitochondrial biogenesis and changes in mitochondrial dynamics, which are aimed at optimizing energy production and preserving cellular functionality (31). Reduced mitochondrial function and disturbance of energy metabolism increases myocardial susceptibility to ischemia-reperfusion injury (IRI) in diabetic hearts. It is interesting that, mitochondrial transplantation (MT) could ameliorate IRI mentioned above, which suggests a whole new treatment strategy (31).

### 2.3 Interaction between mitochondria and carbohydrate metabolism

Mitochondria play a pivotal role in carbohydrate metabolism, especially in relation to glucose utilization and the signaling processes of insulin. Glycolysis, occurring in the cytosol, facilitates the conversion of glucose into pyruvate, which subsequently enters the mitochondria for additional oxidation within the tricarboxylic acid (TCA) cycle. This metabolic pathway is vital for the production of reducing equivalents (NADH and FADH2), which are essential for driving the mitochondrial respiratory chain and facilitating ATP synthesis (31). In conditions characterized by diabetes, the effectiveness of glucose metabolism is frequently hindered due to mitochondrial dysfunction. Specifically, compromised mitochondrial respiration can result in diminished ATP production and an increased dependence on anaerobic glycolysis, which subsequently leads to heightened lactate concentrations and the onset of metabolic acidosis (32). Moreover, the interaction between mitochondrial functionality and insulin signaling pathways is crucial for the maintenance of glucose homeostasis. Dysfunctional mitochondria within insulin-sensitive tissues, such as skeletal muscle and adipose tissue, significantly contribute to insulin resistance and the progression of T2DM (5). Research indicates that enhancing mitochondrial function has the potential to ameliorate glucose metabolism and increase insulin sensitivity. Therapeutic approaches focused on the restoration of mitochondrial health, including the application of mitochondrial-targeted antioxidants or the promotion of mitochondrial biogenesis, have shown encouraging results in improving metabolic outcomes in diabetic models (31). Additionally, the influence of mitochondrial dynamics on carbohydrate metabolism is an emerging field of study, with findings suggesting that modifications in mitochondrial fission and fusion can affect glucose utilization and insulin signaling pathways (32). In summary, the mechanisms underlying mitochondrial energy metabolism are intricate and multifactorial, encompassing the interactions between the mitochondrial respiratory chain, ATP synthesis, and carbohydrate metabolism. A comprehensive understanding of these mechanisms is essential for the formulation of therapeutic interventions aimed at addressing metabolic disorders, particularly in relation to diabetes and other associated conditions. Ongoing research is necessary to clarify the specific molecular pathways implicated in mitochondrial function and their significance for metabolic health.

## 3 The relationship between oxidative stress and mitochondrial function

### 3.1 Sources and effects of oxidative stress

Oxidative stress is characterized as a condition that emerges from the imbalance between the production of reactive oxygen species (ROS) and the organism’s ability to counteract these reactive intermediates or repair the resulting damage. This imbalance can arise from various factors, including exposure to environmental pollutants, inflammatory processes, and metabolic disturbances. In the context of diabetes, oxidative stress is particularly intense, as elevated glucose and fatty acid levels promote the generation of mitochondrial ROS. Mitochondria serve as the primary site for ATP production, while also being the main source of ROS, thus playing a significant role in the oxidative stress paradigm. Increased ROS levels can cause harm to different cellular structures, such as lipids, proteins, and DNA, which contributes to the pathogenesis of diabetes and its related complications (6). The persistent state of oxidative stress not only impairs cellular functions but also triggers inflammatory pathways that further exacerbate tissue damage and dysfunction. For instance, in diabetic conditions, the accumulation of oxidative stress is known to lead to endothelial dysfunction, a precursor to cardiovascular diseases (5). Furthermore, the compromise of antioxidant defenses, evidenced by diminished activity of enzymes such as superoxide dismutase and glutathione peroxidase, intensifies the oxidative environment, thereby creating a vicious cycle of injury and dysfunction (33).

### 3.2 The role of mitochondria in oxidative stress

Mitochondria, as the central organelles of cellular energy metabolism, are not only the primary sites of ROS generation but also crucial regulatory centers for oxidative stress. They are crucial for ATP synthesis via oxidative phosphorylation, while simultaneously acting as significant producers of ROS. The intricate interplay between oxidative stress and mitochondrial dysfunction plays a significant role in various conditions, including aging, neurodegenerative diseases, renal disorders, liver diseases, diabetes, and multiple forms of toxic damage. Under standard physiological circumstances, the generation of ROS occurs as a natural consequence of the electron transport chain’s operation. Nonetheless, in pathological conditions like diabetes, mitochondria exhibit dysfunction, resulting in the excessive generation of ROS, which can overwhelm the cellular antioxidant mechanisms and induce oxidative damage (34). The mitochondrial electron transport chain (ETC) is a critical component of cellular respiration and a primary source of ROS production. Within the ETC, complexes I and III are recognized as significant sites for ROS generation, primarily due to the phenomenon of electron leakage. When electrons are transferred through these complexes, they can escape and react with molecular oxygen, resulting in the formation of superoxide anions, a type of ROS. This leakage is particularly pronounced under conditions of high metabolic demand or when the mitochondrial membrane potential is elevated, leading to increased production of superoxide and other ROS that can contribute to oxidative stress. The reverse electron transfer (RET) mechanism exacerbates ROS generation under specific pathological conditions, such as high substrate availability or when the ETC is impaired. In RET, electrons flow backward through the ETC, particularly from complex II to complex I, which can further increase superoxide production. This phenomenon highlights the delicate balance between mitochondrial function and ROS generation, where normal physiological processes can become pathological under stress conditions. ROS generated during mitochondrial respiration can activate redox-sensitive transcription factors like NF-κB and Nrf2, which in turn modulate gene expression related to antioxidant defenses and cellular metabolism (35).

Mitochondrial-endoplasmic reticulum (ER) interactions play a pivotal role in maintaining cellular homeostasis, particularly in the context of oxidative stress. The contact points between mitochondria and the ER, known as mitochondria-associated membranes (MAMs), serve as critical hubs for Ca^2+^ signaling and ROS production, thereby influencing cellular responses to stress. MAMs facilitate the transfer of Ca^2+^ from the ER to mitochondria, which is essential for ATP production and the regulation of metabolic processes. This Ca^2+^ signaling is crucial, as it can modulate the activity of various mitochondrial enzymes, including those involved in TCA cycle and oxidative phosphorylation, ultimately affecting ROS generation. Elevated ROS levels can lead to oxidative stress, which, if not adequately managed, can result in cellular damage and apoptosis. Conversely, the accumulation of ROS can trigger protective mechanisms, including autophagy, which is vital for the degradation of damaged organelles and the maintenance of cellular integrity (36).

The balance between mitochondrial fusion and fission is essential for maintaining mitochondrial integrity and function. Disruption of this balance can lead to mitochondrial dysfunction, characterized by excessive ROS production, which has been implicated in various pathologies, including neurodegenerative diseases and aging (35). The synthesis of respiratory chain complexes, including those that form the respiratory chain complexes I-IV and ATP synthase, essential for ATP production, relies heavily on the integrity of mtDNA, the damage of mtDNA is a critical consequence of oxidative stress, primarily induced by ROS. The mitochondrial dysfunction observed in diabetic individuals is marked by alterations in mitochondrial morphology, reduced bioenergetic capacity, and compromised mitophagy, which can selectively degrades damaged or dysfunctional mitochondria, and maintain mitochondrial quality and cellular homeostasis.The PINK1 (PTEN-induced putative kinase 1)/Parkin pathway is one of the most well-characterized mechanisms of mitophagy. PINK1 accumulates on the outer mitochondrial membrane of depolarized mitochondria, where it phosphorylates and activates Parkin, an E3 ubiquitin ligase. This activation leads to the ubiquitination of various mitochondrial proteins, marking them for degradation by the autophagy machinery (37, 38). In response to increased glucose levels in blood, pancreatic β-cells exhibit glucose uptake and metabolism to generate ATP, that triggers depolarization of the plasma membrane, influx of extracellular Ca^2+^, and insulin secretion to reduce blood glucose levels (39). Mitochondrial dysfunction in pancreatic B-cells leads to impaired glucose-stimulated insulinsecretion (GSIS) and T2D, highlighting the importance of autophagic elimination of mitophagy in mitochondrial quality control (mQC). Mitochondrial dysfunction is associated with an increased vulnerability to oxidative stress and plays a role in the progression of diabetic complications such as diabetic cardiomyopathy and nephropathy (32). Furthermore, disruptions in mitochondrial dynamics, including the processes of fission and fusion, aggravate oxidative stress by facilitating the accumulation of dysfunctional mitochondria, which in turn leads to heightened ROS production (5). Consequently, the preservation of mitochondrial integrity is vital for alleviating oxidative stress and its adverse impacts on cellular functionality.

### 3.3 The impact of oxidative stress on insulin signaling

Oxidative stress significantly influences insulin signaling pathways, which are essential for maintaining glucose balance. In tissues that are sensitive to insulin, such as skeletal muscle and adipose tissue, oxidative stress can disrupt insulin receptor signaling, resulting in insulin resistance (40). The accumulation of ROS has the potential to activate kinases sensitive to stress, which in turn phosphorylate IRS. This phosphorylation leads to the degradation of IRS and subsequently hinders downstream signaling pathways, notably the PI3K/Akt pathway (32). Such disruption culminates in a diminished translocation of glucose transporter type 4 (GLUT4) to the plasma membrane, consequently lowering glucose uptake and contributing to elevated blood sugar levels. Additionally, oxidative stress can provoke inflammatory responses, further worsening insulin resistance through the activation of pro-inflammatory cytokines that disrupt insulin signalling (32). In diabetic models, strategies aimed at reducing oxidative stress have demonstrated an enhancement in insulin sensitivity, underscoring the necessity of addressing oxidative stress in the management of diabetes (40). Therefore, comprehending the intricate relationship between oxidative stress and insulin signaling is vital for devising therapeutic approaches focused on restoring insulin sensitivity and enhancing metabolic health in diabetic patients.

## 4 Genetic susceptibility to diabetes

### 4.1 Genetic mutations affecting mitochondrial function

DM is a complex metabolic disorder influenced by a multitude of genetic and environmental factors. DM has a significant genetic predisposition, and children of both parents with diabetes are 15 to 20 times more likely to develop diabetes (41). The clinical heterogeneity of diabetes is further complicated by its genetic underpinnings. A crucial element in the pathophysiology of diabetes is its inherent genetic predisposition, which has gained prominence as a key determinant in the initiation and advancement of the disease. According to the genetic pattern and the mechanism of action, DM can be divided into monogenic diabetes and polygenic diabetes. Monogenic diabetes, though rare, has been identified in various forms, each with distinct genetic mutations affecting insulin secretion and action, with notable examples including GCK, HNF1A, HNF4A, HNF1B, and PDX1. Genetic polymorphisms, especially those that influence mitochondrial functionality and insulin signaling mechanisms, are integral to the etiology of diabetes (42). Compared with the nuclear genome, naked histone-lacking mtDNA exhibits higher mutation rates due to fewer DNA repair mechanisms in the mitochondrial genome, the lack of proofreading capability of the mtDNA polymerase, and the lack of histone shielding (43). Mutations in mtDNA can interfere with the oxidative phosphorylation process, leading to diminished ATP synthesis and an increase in ROS production. Such modifications can instigate a series of cellular responses that culminate in insulin resistance and beta-cell impairment, ultimately facilitating the onset of T2DM (44). A3243G mutation in mtDNA–encoded tRNA^Leu(UUR)^ gene is associated with maternally inherited diabetes and deafness (MIDD). The glucose-stimulated insulin secretion, mitochondrial functionality and energy metabolism of patients carried 3243 mutation was reduced significantly, increasing the risk of developing diabetes (45). Mutant mitochondrial tRNA^Leu^ is less efficiently aminoacylated than wild-type tRNA. Furthermore, the balance between mitochondrial and nuclear-encoded proteins in mutant mitochondria was altered, leading to mitochondrial dysfunction (46, 47). In addition, mutations that impact genes responsible for mitochondrial dynamics—specifically those coding for proteins that modulate mitochondrial fission and fusion—have been associated with complications arising from diabetes. For instance, changes in the expression levels of dynamin-related protein 1 (DRP1) and mitofusins may result in compromised mitochondrial structure and functionality, which are hallmark features of diabetic conditions (48). The interaction between genetic mutations and environmental influences, such as hyperglycemia, further complicates the connection between mitochondrial dysfunction and diabetes. Prolonged hyperglycemic states can exacerbate mitochondrial injury and mutations in mtDNA, leading to a detrimental cycle of oxidative stress and metabolic imbalance. This underscores the necessity of elucidating the genetic foundations of mitochondrial dysfunction within the framework of diabetes, as it may unveil potential therapeutic avenues aimed at reinstating mitochondrial integrity and enhancing metabolic outcomes (49).

### 4.2 Genetic mutations leading to the pathogenesis of DM

The genetic components underlying diabetes encompass a variety of mutations that can manifest as both monogenic and polygenic forms of the condition. Monogenic diabetes, although representing a minor proportion of overall diabetes cases, is primarily attributed to mutations in individual genes vital for insulin production or its action. For instance, mutations in the insulin gene (INS) can result in neonatal diabetes mellitus, which is characterized by the presence of hyperglycemia within the initial six months of life (50). R6C/H lowers the production of insulin by ~50 percent and contribute to the development of MIDY and late-onset diabetes (51). The preproinsulin-A (SP24)D mutation blocks ER exit and accelerates the development of MIDY (52). R89C could affect the proteolytic processing of proinsulin in ER, introducing an additional cysteine residue and cause permanent neonatal diabetes mellitus. Mutations of MAFA and MAFB cause overt dysfunction that leads to T2DM (53). The G32S mutation affects insulin biosynthesis, thus contributing to the development of neonatal- and infancy-onset diabetes, is considered an important biomarker for the diagnosis of neonatal- and infancy-onset diabetes (54). L30M affects coding and insulin multimerization, R46Q mutation affects insulin biosynthesis. They could compromise the activity and function of β-cells, and lead to the development of MODY (55). F49L leads to hyperinsulinemia and hyperproinsulinemia in patients while F48S leads to the production of structurally abnormal insulin. V92L affects the biosynthesis of normal insulin, weakens receptor binding and biological activity (56, 57). In a word, mutations of INS mentioned above typically lead to impaired insulin production, culminating in significant metabolic disruptions.

In addition to monogenic diabetes, considerable research has been conducted on the polygenic predisposition to T2DM. Genome-wide association studies (GWAS) have revealed a multitude of genetic variants correlated with an increased likelihood of developing T2DM. The genetic landscape of T2D is complex, involving numerous genetic variants that contribute to the disease’s pathophysiology. These variants frequently influence pathways related to insulin signaling, glucose metabolism, and lipid regulation. For example, polymorphisms in genes such as TCF7L2, FTO, and KCNJ11 have been consistently associated with a heightened risk of T2DM across various demographic groups (58). One of the key players in this genetic framework is the IRS1 gene, whose variants have been implicated in insulin signaling abnormalities. Variants in IRS1 can disrupt the insulin signaling pathway, leading to insulin resistance, a hallmark of T2D. A study identified a likely pathogenic missense mutation in IRS1 (c.2137C > T, p.His713Tyr) that co-segregated with early-onset T2D in a family, highlighting the gene’s role in the disease’s etiology. It is of importance to screen at the genomic level in identifying individuals at risk for T2D, particularly those with a family history of early-onset diabetes (59). The additive effects of these genetic variations, in conjunction with environmental influences such as dietary habits and physical activity, contribute to the intricate etiology of T2DM. Moreover, recent investigations have emphasized the significance of epigenetic alterations and gene-environment interactions in the predisposition to diabetes. Elements such as maternal dietary habits during gestation and early-life environmental exposures can modulate gene expression, thereby impacting the likelihood of developing diabetes in later stages of life. This highlights the necessity for a comprehensive approach to deciphering the genetic foundations of diabetes, which integrates genetic, epigenetic, and environmental aspects to clarify the mechanisms that contribute to the disease (60). In summary, the genetic vulnerability to diabetes is a multifaceted phenomenon shaped by a variety of genetic mutations influencing mitochondrial functions and insulin signaling pathways. Gaining insights into these genetic foundations is essential for crafting targeted interventions and personalized treatment approaches aimed at effectively preventing and managing diabetes. Continued exploration of the complex interplay between genetic mutations, mitochondrial dysfunction, and the onset of diabetes will be vital for enhancing our understanding and improving clinical outcomes for those at risk of this widespread condition.

## 5 Mitochondrial dysfunction and insulin resistance

### 5.1 Mitochondrial dynamics and insulin sensitivity

Mitochondrial dysfunction has emerged as a critical factor in the development of insulin resistance, especially within the framework of metabolic conditions like T2DM. The complex interplay between mitochondrial integrity and insulin sensitivity has attracted considerable scrutiny in contemporary research, demonstrating that compromised mitochondrial functionality can initiate a series of metabolic disruptions leading to insulin resistance. Mitochondrial dynamics, encompassing processes such as fusion, fission, and mitophagy, are pivotal for sustaining mitochondrial functionality and ensuring cellular energy equilibrium. In tissues that exhibit sensitivity to insulin, including skeletal muscle and adipose tissue, the regulation of mitochondrial dynamics is crucial for optimal insulin signaling and glucose metabolism. Research has demonstrated that in the presence of insulin resistance, there tends to be a transition towards fragmented and dysfunctional mitochondrial networks, marked by an increase in fission and a decrease in fusion events (61). This fragmentation correlates with diminished oxidative phosphorylation capacity and compromised ATP synthesis, both of which are vital for preserving insulin sensitivity. The significance of mitochondrial dynamics in the context of insulin sensitivity is further corroborated by studies indicating that enhanced mitochondrial fission, mediated by proteins like Drp1, can instigate elevated production of ROS and subsequent oxidative stress, both of which adversely affect insulin signaling pathways (62). On the other hand, the promotion of mitochondrial fusion via the activation of proteins such as Mfn1 and Mfn2 has been observed to enhance mitochondrial function and improve insulin sensitivity. This indicates that therapeutic interventions focused on restoring mitochondrial dynamics may hold promise in addressing insulin resistance. Furthermore, the interplay between mitochondria and other organelles, notably the ER, is essential for the maintenance of cellular homeostasis. MAMs facilitate the communication between mitochondria and the ER, playing a crucial role in lipid metabolism and calcium signaling. The disruption of MAM integrity has been associated with insulin resistance, underscoring the significance of inter-organelle communication in metabolic health (63). Consequently, a thorough understanding of mitochondrial dynamics and their interactions with various cellular components may unveil novel strategies for enhancing insulin sensitivity in individuals suffering from metabolic disorders.

### 5.2 Mitochondrial gene expression and insulin resistance

The expression of mitochondrial genes is essential for sustaining mitochondrial functionality and energy metabolism. In states characterized by insulin resistance, variations in the expression of genes that encode mitochondrial proteins can result in compromised oxidative phosphorylation and an elevation in (ROS production, which collectively contribute to the progression of insulin resistance. Research has indicated that mutations in mtDNA and epigenetic modifications can adversely affect the expression of critical mitochondrial genes, thus disrupting mitochondrial function and fostering insulin resistance (64).

Evidence points to a correlation between insulin resistance and the modified expression of genes implicated in mitochondrial biogenesis, including PGC-1α, NRF1, and TFAM, all of which are vital for preserving mitochondrial mass and performance (32). In individuals exhibiting insulin resistance, a notable decline in the levels of these transcription factors is observed, which consequently leads to diminished mitochondrial biogenesis and functionality. This decrease in mitochondrial capacity is further intensified by heightened lipotoxicity and glucotoxicity, which can compromise mitochondrial components and hinder their operational efficacy (33).

Furthermore, the expression of genes associated with ETC is frequently suppressed in tissues affected by insulin resistance, resulting in reduced ATP production and heightened oxidative stress (5). This detrimental cycle of mitochondrial dysfunction and insulin resistance underscores the necessity for therapeutic approaches aimed at reinstating mitochondrial gene expression and functionality. Strategies that promote mitochondrial biogenesis and enhance mitochondrial gene expression may offer promising avenues for reversing insulin resistance and bolstering metabolic health.

### 5.3 Findings from animal models

Animal models have played a pivotal role in clarifying the connection between mitochondrial dysfunction and insulin resistance. Numerous investigations employing rodent models of insulin resistance have indicated that mitochondrial impairment occurs prior to the manifestation of insulin resistance and is a crucial element in its progression (65, 66). For instance, in models of obesity induced by high-fat diets, mitochondrial dysfunction has been associated with heightened insulin resistance and diminished glucose tolerance (67).

In these experimental setups, strategies aimed at enhancing mitochondrial function—such as exercise interventions or pharmacological agents designed to boost mitochondrial biogenesis—have been shown to restore insulin sensitivity (31). Moreover, research utilizing mitochondrial transplantation techniques has demonstrated that the reintroduction of healthy mitochondria can alleviate insulin resistance and enhance metabolic outcomes in diabetic animal models (68). These observations highlight the therapeutic potential of targeting mitochondrial health as a means to prevent and manage insulin resistance and associated metabolic disorders. In addition, genetic modifications in animal models that improve mitochondrial dynamics or encourage mitochondrial biogenesis have shed light on the mechanisms through which mitochondrial function affects insulin sensitivity. For example, mice exhibiting increased expression of genes related to mitochondrial biogenesis show improved insulin sensitivity and metabolic profiles, thereby emphasizing the significance of mitochondrial health in glucose metabolism regulation (69).

In conclusion, the relationship between mitochondrial function, gene expression, and insulin resistance is intricate and multifaceted. Gaining a deeper understanding of these associations through animal models offers crucial insights into potential therapeutic strategies focused on enhancing mitochondrial health and restoring insulin sensitivity in individuals affected by metabolic disorders. As research progresses in uncovering the complex interactions between mitochondria and insulin resistance, novel approaches may emerge to address the escalating prevalence of T2DM and related health conditions.

## 6 Interaction between mitochondria and inflammatory responses

### 6.1 Impact of inflammatory cytokines

Inflammatory cytokines, including tumor necrosis factor-alpha (TNF-α), interleukin-6 (IL-6), and interleukin-1 beta (IL-1β), are crucial in the development of numerous diseases due to their influence on mitochondrial function. These cytokines can trigger mitochondrial dysfunction, resulting in heightened oxidative stress and compromised bioenergetics. For example, TNF-α has been demonstrated to facilitate mitochondrial fragmentation and disrupt mitochondrial dynamics, which may aggravate inflammation and contribute to tissue injury (70). The activation of the NLRP3 inflammasome by mitochondrial damage-associated molecular patterns (DAMPs) further underscores the relationship between mitochondrial dysfunction and the inflammatory response (71). Additionally, the release of mtDNA into the cytosolic environment can initiate the cGAS-STING signaling pathway, leading to the generation of type I interferons and further inflammatory mediators (72). This indicates that the interplay between inflammatory cytokines and mitochondria is reciprocal; cytokines can provoke mitochondrial stress, while dysfunctional mitochondria can enhance inflammatory signaling.

### 6.2 Role of mitochondria in inflammatory pathways

Mitochondria are increasingly acknowledged as vital contributors to the regulation of inflammatory processes, affecting both the onset and the resolution of inflammation. Their functions encompass more than mere energy production, extending to the modulation of immune responses and the synthesis of ROS, which serve as signaling entities within inflammatory pathways. Impairments in mitochondrial function have been associated with numerous inflammatory diseases, such as cardiovascular ailments, neurodegenerative conditions, and metabolic syndromes. For instance, the production of ROS by mitochondria is a significant contributor to the activation of NF-κB and other transcription factors that participate in inflammatory processes (73). In macrophages, the dynamics of mitochondria, characterized by fission and fusion events, are vital for their activation in a pro-inflammatory context. An increase in mitochondrial mass and fragmentation has been linked to a heightened release of inflammatory cytokines in response to stimuli such as lipopolysaccharides (LPS) (74). Additionally, the relationship between mitochondrial metabolism and immune cell functionality is particularly evident during the metabolic reprogramming that occurs in inflammation. For example, the transition from oxidative phosphorylation to glycolysis in activated macrophages is facilitated by mitochondrial signaling, which meets the energy requirements of pro-inflammatory activities (75). This metabolic adaptability highlights the crucial role of mitochondria in influencing the inflammatory milieu.

### 6.3 Prospects of mitochondrial-targeted therapies

Given the crucial involvement of mitochondria in the inflammatory process, the strategic targeting of these organelles emerges as a promising therapeutic approach for addressing inflammatory disorders. Therapeutic strategies aimed at mitochondria seek to restore their functionality, diminish oxidative stress, and modulate inflammatory processes. For instance, the application of mitochondrial antioxidants has demonstrated potential in alleviating inflammation by effectively scavenging ROS and averting oxidative damage (76). Furthermore, methodologies that promote mitophagy may contribute to the preservation of mitochondrial integrity and the attenuation of inflammation in conditions such as diabetic cardiomyopathy (32). Novel therapies, including mitochondria-targeted nanoparticles and pharmaceutical agents, are currently being developed to deliver therapeutic compounds directly to the mitochondria, thereby enhancing their therapeutic efficacy while minimizing systemic adverse effects (77). These pioneering strategies hold considerable promise for the treatment of various inflammatory diseases by harnessing the distinctive characteristics of mitochondria to modulate immune responses and restore physiological equilibrium. In summary, the relationship between mitochondria and inflammatory responses is intricate and multifaceted. Mitochondria not only impact the production of inflammatory cytokines but also play essential roles in regulating immune cell activities. A deeper understanding of these interactions paves the way for new therapeutic interventions designed to alleviate inflammation and its related complications. As ongoing research continues to uncover the sophisticated roles of mitochondria in inflammation, the advancement of targeted therapies may offer effective solutions for managing inflammatory diseases and enhancing patient outcomes.

## 7 Emerging therapies and mitochondrial protection

### 7.1 Application of antioxidants and calcium homeostasis

The significance of antioxidants in safeguarding mitochondrial health has attracted considerable scholarly interest, primarily due to their capability to alleviate oxidative stress, a fundamental factor implicated in mitochondrial impairment and a spectrum of diseases such as diabetes and neurodegenerative conditions. Antioxidants are adept at neutralizing ROS that are generated during cellular metabolic processes, thereby maintaining the structural and functional integrity of mitochondria. Mitochondria are the primary source of ROS in cells, and their regulation is crucial for maintaining cellular health. Antioxidants, both endogenous and exogenous, play a vital role in mitigating oxidative stress by neutralizing ROS and preventing cellular damage. Recent studies have highlighted the potential of mitochondria-targeted antioxidants, such as triphenylphosphonium (TPP)-conjugated compounds, which exhibit significantly enhanced accumulation within mitochondria due to the organelle’s negative membrane potential (78). Mitochondrial-targeted antioxidants, including MitoQ and SkQ1, are proficient in diminishing oxidative harm within mitochondria, consequently enhancing cellular vitality and functionality (79). These agents are engineered to accumulate within the mitochondria, where they perform their protective roles by scavenging ROS and improving mitochondrial bioenergetic processes. Moreover, the incorporation of dietary antioxidants, such as vitamins C and E, has been associated with enhanced mitochondrial function and diminished oxidative stress across various experimental models, underscoring the significance of dietary strategies in fostering mitochondrial wellness (80). Additionally, the innovation of novel antioxidants that specifically interact with mitochondrial pathways shows promise for the treatment of disorders characterized by mitochondrial dysfunction, including T2DM and Alzheimer’s disease (81). In summary, the utilization of antioxidants emerges as a vital approach within the burgeoning field of mitochondrial protection, with far-reaching implications for a diverse array of metabolic and degenerative ailments.

Moreover, the interplay between ROS and calcium homeostasis is critical. Ca²^+^ serve as essential second messengers in numerous signaling pathways, including those governing cell survival and apoptosis. Dysregulation of calcium homeostasis can exacerbate oxidative stress, leading to a vicious cycle of cellular damage. Therefore, strategies aimed at restoring both antioxidant defenses and calcium homeostasis are emerging as promising therapeutic approaches for treating oxidative stress-related diseases. Maintaining calcium homeostasis is equally crucial for preventing cellular damage and ensuring proper cellular function. Ca²^+^ are vital for various physiological processes, including muscle contraction, neurotransmitter release, and cell signaling. In the context of mitochondrial function, calcium uptake into mitochondria is essential for ATP production and metabolic regulation. However, excessive calcium influx can lead to mitochondrial overload, resulting in oxidative stress and cell death. This phenomenon is particularly relevant in conditions such as ischemia-reperfusion injury, where calcium overload contributes to neuronal cell death (82). Recent research has shown that modulating calcium levels can influence mitochondrial function and cellular outcomes. For instance, the activation of calcium-sensing receptors and channels can enhance calcium influx, which in turn may promote protective signaling pathways that counteract oxidative stress. Conversely, inhibiting calcium channels has been shown to reduce oxidative stress and improve cell survival in models of neurodegeneration and ischemia (83).

Understanding the intricate relationship between calcium homeostasis and oxidative stress is vital for developing effective therapeutic strategies. Interventions that target both antioxidant pathways and calcium regulation may provide synergistic benefits, enhancing cellular resilience against oxidative damage and improving outcomes in various diseases linked to mitochondrial dysfunction.

### 7.2 Mitochondria-mediated drug development

Mitochondria have increasingly been identified as crucial targets in the realm of drug development, especially in relation to disorders characterized by mitochondrial dysfunction, including diabetes, cancer, and neurodegenerative diseases. Mitochondrial-targeted drug delivery has emerged as a promising strategy in pharmacotherapy. One of the key methodologies for achieving selective mitochondrial targeting involves the use of lipophilic cations, such as triphenylphosphonium (TPP) and dequalinium (DQA), which exploit the negative mitochondrial membrane potential to facilitate the accumulation of therapeutic agents within these organelles. Recent progresses in pharmaceutical design have concentrated on formulating compounds that specifically engage mitochondrial pathways, thereby enhancing therapeutic effectiveness while reducing systemic adverse effects. For example, the creation of mitochondria-targeted therapies, such as small molecules capable of selectively triggering mitochondrial apoptosis in cancerous cells, has demonstrated encouraging results in preclinical investigations (84). These pharmacological agents capitalize on the distinct metabolic properties of cancer cells, which frequently depend on mitochondrial activity for energy generation and survival. Additionally, innovative methodologies involving mitochondrial transplantation have emerged, where healthy mitochondria are introduced into cells exhibiting compromised mitochondrial function, revealing potential applications in the treatment of conditions like diabetic cardiomyopathy (68). This cutting-edge approach aims not only to restore energy production but also to bolster cellular resilience against various stressors. Moreover, the incorporation of nanotechnology into drug delivery systems facilitates the targeted administration of therapeutics to mitochondria, thereby enhancing the pharmacological efficacy against diseases associated with mitochondrial dysfunction (85).

Recent advancements in nanotechnology have opened new avenues for enhancing the delivery and efficacy of mitochondria-targeted therapies. Nanoparticles engineered for mitochondria-specific delivery can improve the bioavailability of drugs, facilitate controlled release, and enhance therapeutic outcomes (86). Various strategies, including the use of lipophilic cations, mitochondrial-penetrating peptides, and the development of multifunctional nanocarriers, are being explored to optimize drug delivery to mitochondria. These innovations are critical for addressing the limitations of conventional drug therapies and represent a significant step forward in the field of targeted drug development.

Drug resistance, particularly in the context of cancer therapies, has emerged as a significant obstacle to effective treatment. One of the molecular mechanisms contributing to drug resistance is the mutation of mitochondrial genes. mtDNA mutations have been implicated in altering the metabolic pathways of cancer cells, thereby enabling them to survive in the presence of chemotherapeutic agents. Alterations in the mitochondrial respiratory chain can lead to increased ROS, and promote cell survival and resistance to apoptosis induced by conventional therapies. Especially in breast and ovarian cancer, mutations in mtDNA have been linked to poor prognosis and treatment outcomes (87). To combat drug resistance, innovative therapeutic strategies have been developed, including multi-target combination therapies and triplet therapy approaches. Multi-target combination therapies aim to simultaneously inhibit multiple pathways that cancer cells exploit for survival. For example, combining mitochondrial-targeting agents with traditional chemotherapeutics can enhance the overall therapeutic efficacy by addressing the various mechanisms of resistance. Recent studies have demonstrated that such combination strategies can significantly reduce the viability of drug-resistant cancer cells by overwhelming their adaptive responses. Furthermore, triplet therapy, which involves the use of three different drugs targeting distinct vulnerabilities within cancer cells, has shown promise in preclinical models. This approach not only reduces the likelihood of resistance development but also enhances the overall treatment response by attacking the cancer from multiple angles (88).

Together, these advancements highlight the significance of mitochondria as a therapeutic target and the potential for mitochondria-focused drug development to transform treatment paradigms for a range of diseases. The clinical translation of mitochondria-targeted therapies poses significant challenges, primarily due to issues surrounding carrier biocompatibility, immune responses, and *in vivo* distribution. Mitochondria-targeted drug delivery systems must navigate the complexities of biological environments, where the stability and efficacy of therapeutic agents can be compromised by the host’s immune response. The use of nanoparticles or lipophilic cations as carriers can elicit unwanted immune reactions, leading to rapid clearance from circulation and reduced therapeutic efficacy (89). Moreover, achieving precise localization of drugs within mitochondria is complicated by the dynamic nature of these organelles and their interaction with various cellular components. Strategies to enhance biocompatibility include the use of biodegradable materials and the incorporation of targeting ligands that can facilitate selective uptake by cells while minimizing off-target effects (90).

### 7.3 Therapeutic of DM via targeting mitochondrial

**Figure 2:**
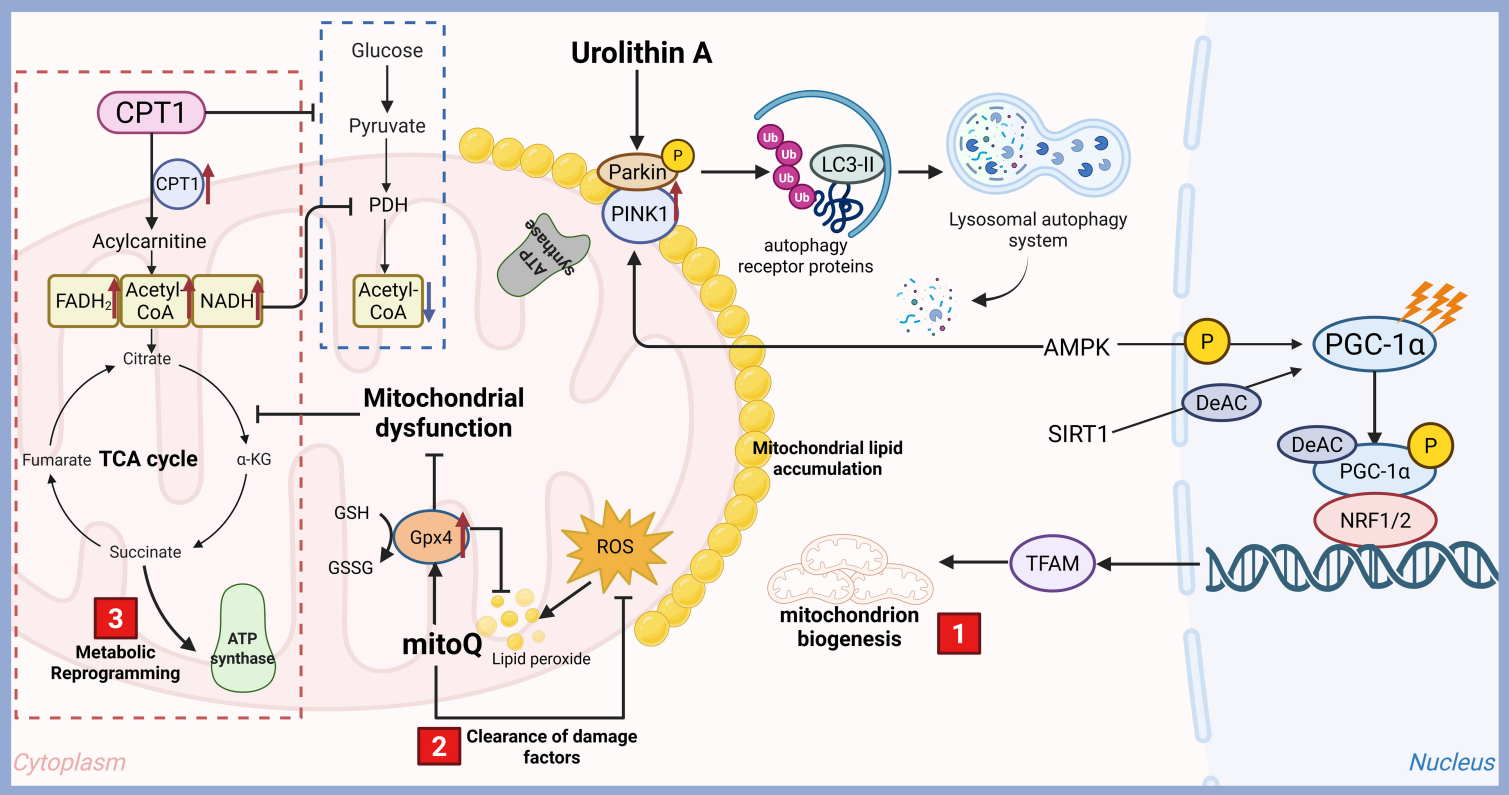
Therapeutic targets for diabetes targeting mitochondrial function. Mitochondria are double-membrane-bound organelles essential for cellular energy production and metabolic homeostasis. Their functions extend far beyond ATP synthesis, encompassing critical roles in signaling, cell death regulation, and metabolic integration, which are more or less directly or indirectly related to DM. With the rapid development of technology, many new therapeutic targets for DM have emerged, including mitochondrial biogenesis and function improving, metabolic reprogramming and clearance of damage factors.

Mitochondria are not merely “powerhouses” but dynamic signaling organelles that regulate metabolism, cell death, redox balance, and calcium signaling, which are more or less directly or indirectly related to DM. Many new therapeutic targets for DM have emerged (**Figure 2**). Metformin, AICAR and berberine can activate AMPK through increasing PGC-1α expression, mitochondrial biogenesis, FAO and glucose uptake will be boosted (91). GW501516, a PPARδ agonists, can stimulate PGC-1α which is a master regulator of mitochondrial biogenesis and improve oxidative capacity (92). Nicotinamide riboside and nicotinamide mononucleotide can booster NAD^+^and activate SIRT1/3, enhance biogenesis and FAO via deacetylating PGC-1α/ETC proteins (93). Mdivi-1 (Drp1 inhibitors) and Mfn2 agonists can improve fission/fusion balance of mitochondria and reduce fragmented mitochondria through inhibiting excessive fission or promoting fusion (94). Urolithin A and rapamycin can enhance mitophagy to clear damaged mitochondria via PINK1/Parkin or BNIP3 pathways (95). Mitochondrial antioxidants, such as MitoQ, SkQ1 and SS-31, can scavenge ROS directly in mitochondria to combate oxidative stress (96). Sulforaphane and bardoxolone methyl, Nrf2 activators, can upregulate antioxidant genes, such as SOD2 and glutathione, reduce oxidative damage (97). Piromelatine and malonyl-CoA decarboxylase inducers are all CPT1 modulators which can promote fatty acid entry into mitochondria for β-oxidation (98). Dichloroacetate, pyruvate dehydrogenase (PDH) activator, is able to shift metabolism from glycolysis to OXPHOS. CPT1 Modulators and PDH activator all can optimize substrate utilization of mitochondria (99). Low-dose 2,4-dinitrophenol can reduce ROS, improve glucose uptake via UCP1/2 activation or chemical uncouplers (100). Anti-apoptotic agents (Cyclosporin A, sanglifehrin A) and mKATP channel modulators (Diazoxide) can protecte β-Cell mitochondria via blocking mitochondrial permeability transition pore (mPTP), regulating mitochondrial membrane potential to prevent Ca²^+^ overload/ROS in β-cells respectively (101). In conclusion, mitochondrial targeting represents a paradigm shift in diabetes treatment, moving beyond symptomatic control to address root metabolic dysfunction.

### 7.4 Impact of lifestyle interventions on mitochondrial function

Lifestyle modifications, encompassing alterations in diet and increased physical activity, exert significant effects on mitochondrial functionality and overall metabolic wellness. Engaging in regular exercise has been demonstrated to boost mitochondrial biogenesis, enhance oxidative capacity, and optimize energy metabolism efficiency across various tissues, particularly in skeletal muscle (102). Notably, high-intensity interval training (HIIT) has been correlated with remarkable improvements in mitochondrial performance, facilitating adaptations that promote fat oxidation and enhance insulin sensitivity (103). Likewise, dietary strategies such as caloric restriction and adherence to a plant-based diet abundant in antioxidants have been associated with enhanced mitochondrial health and diminished oxidative stress, further contributing to metabolic equilibrium (104). Additionally, burgeoning evidence indicates that time-restricted eating may positively affect mitochondrial dynamics and functionality, potentially providing a novel strategy to counteract age-related declines in mitochondrial efficiency (105). These lifestyle alterations not only foster mitochondrial well-being but also act as proactive measures against chronic conditions, including T2DM, cardiovascular diseases, and neurodegenerative disorders. As investigations continue to clarify the mechanisms through which lifestyle modifications influence mitochondrial function, there is an increasing acknowledgment of their potential as efficacious therapeutic approaches to promote health and longevity.

## 8 Conclusion

In recent years, there has been a substantial increase in the recognition of mitochondrial dysfunction as a pivotal factor in the development of diabetes. Mitochondria serve as the cell’s energy generators, facilitating energy production through oxidative phosphorylation, while also playing essential roles in the modulation of oxidative stress and insulin sensitivity. As discussed in this review, the diverse functions of mitochondria in energy metabolism, cellular signaling, and apoptosis underscore their significance in both the initiation and advancement of diabetes.

The repercussions of mitochondrial dysfunction on diabetes manifest in various aspects, including disrupted glucose homeostasis, heightened oxidative stress, and the emergence of insulin resistance. Collectively, these elements contribute to the metabolic syndrome, along with its related complications. The interplay between energy metabolism and oxidative stress further complicates the situation, as the elevated production ofROSis both a consequence of mitochondrial dysfunction and a factor that aggravates metabolic disturbances, establishing a challenging cycle that is difficult to disrupt.

Considering this complex relationship, there is an increasing interest in mitochondrial protection and repair as a promising therapeutic strategy for managing diabetes. Approaches designed to enhance mitochondrial function, such as the application of antioxidants, stimulators of mitochondrial biogenesis, and compounds that improve mitochondrial dynamics, have demonstrated potential in preclinical studies. Importantly, these interventions not only address the metabolic dysfunction linked to diabetes but also seek to reduce oxidative stress, thereby targeting two fundamental aspects of the disease’s pathology.

However, as we advance in this field, it is crucial to balance the various research perspectives. Although mitochondrial dysfunction remains a prominent focal point, it is vital to recognize the multifactorial aspects inherent in diabetes. Factors such as genetic predispositions, environmental influences, and lifestyle decisions significantly contribute to the onset and advancement of this condition. Consequently, forthcoming investigations should aspire to adopt a comprehensive approach that interlinks mitochondrial dynamics with additional metabolic pathways and systemic elements. This strategy would facilitate the formulation of thorough treatment protocols that not only target mitochondrial functions but also account for the wider metabolic landscape.

Furthermore, translating fundamental mitochondrial research into clinical applications necessitates an exhaustive examination of the precise mechanisms through which mitochondrial dysfunction influences diabetes. Gaining insights into these pathways will be essential for devising targeted pharmacological therapies and lifestyle adjustments that can effectively restore mitochondrial efficiency and enhance metabolic health.

In summary, investigating mitochondrial functionality in the context of diabetes heralds a promising avenue for therapeutic innovation. As we progressively decode the intricacies of mitochondrial participation in metabolic disorders, it is crucial to uphold a balanced viewpoint that acknowledges the interconnections among various biological systems. Collaborative initiatives among researchers, healthcare professionals, and pharmaceutical innovators will be vital in propelling forward groundbreaking solutions that leverage mitochondrial biology to effectively address diabetes. Ultimately, the aim is to translate these findings into clinically applicable interventions that can enhance patient outcomes and elevate the quality of life for individuals affected by diabetes. As we gaze toward the future, continuous research will inevitably uncover novel pathways and mechanisms, thereby paving the way for innovative therapeutic approaches designed to alleviate the burden of diabetes on patients and healthcare systems alike.
